# Neurocognitive function in patients at high risk of schizophrenia with positive thought disorders

**DOI:** 10.1192/j.eurpsy.2021.1401

**Published:** 2021-08-13

**Authors:** I. Fateev, M. Omelchenko, I. Pluzhnikov

**Affiliations:** 1 Department Of Youth Psychiatry, The Mental Health Research Center, Moscow, Russian Federation; 2 Department Of Adult Neuropsychology And Abnormal Psychology, Moscow Institute of Psychoanalysis, Moscow, Russian Federation

**Keywords:** Positive thought disorder, high risk of schizophrenia, Neurocognitive function, inhibitory control

## Abstract

**Introduction:**

The course of affective disorders varies significantly in clinical practice. There are many symptoms that are not related to affective disorders that cannot be described in other nosologies. In the present study such pathopsychological phenomena similar to psychotic symptoms and related to symptoms of “schizophrenia risk” were designated as positive thought disorders (PTD). These symptoms are understood as manifestations of delusional and hallucinatory register.

**Objectives:**

Aim of the study is to identify and validate the differences of neurocognitive functions among patients with positive thought disorders and at high risk of schizophrenia and patients without thought disorders.

**Methods:**

In the research there were 17 patients with high risk of schizophrenia dominated by PTD (affective disorders, personality disorders, schizophrenic spectrum disorders) and 18 patients without thought disorders (affective disorders, personality disorders) in the research. Patients aged 17-25 years.

**Results:**

According to the results of the The Complex Figure test, the group with a high risk of schizophrenia had significantly low results on the “simultaneity” scale and points for copying the figure (p-value 0.04 and p-value 0.03). According to the results of the Verbal fluency test, the main group had significantly lower indices on the “loss of instruction” scale and on the number of repetitions (p-value 0.021 and p-value 0.009).

**Conclusions:**

In the group of patients with a high risk of schizophrenia with positive thought disorders there are neurocognitive features in the form of reduced inhibitory control and a lack of simultaneity. The most sensitive methods are the Complex figure test and Verbal fluency Test.

**Conflict of interest:**

The reported study was funded by RFBR, project number 20-013-00772

